# Pesticide Residues and Berry Microbiome after Ozonated Water Washing in Table Grape Storage

**DOI:** 10.3390/foods12173144

**Published:** 2023-08-22

**Authors:** Gabriele Caponio, Marco Vendemia, Domenica Mallardi, Antonio Domenico Marsico, Vittorio Alba, Giovanni Gentilesco, Giovanna Forte, Riccardo Velasco, Antonio Coletta

**Affiliations:** CREA, Council for Agricultural Research and Economics, Research Center for Viticulture and Enology, 70010 Turi, Italy; gabriele.caponio@crea.gov.it (G.C.); marco.vendemia@crea.gov.it (M.V.); domenica.mallardi@crea.gov.it (D.M.); adomenico.marsico@crea.gov.it (A.D.M.); vittorio.alba@crea.gov.it (V.A.); giovanni.gentilesco@crea.gov.it (G.G.); giovanna.forte@crea.gov.it (G.F.); riccardo.velasco@crea.gov.it (R.V.)

**Keywords:** ozonated water, pesticide, grape, postharvest, storage

## Abstract

Nowadays, different systems for reducing pesticides in table grapes are being tested at different production stages either in the field or in postharvest. The present study tested ozonated water treatments at the beginning of the cold storage of the Princess^®^ seedless table grape variety to reduce the residue contents of some pesticides and to evaluate their effect on gray mold and the berry microbiome. An ozone generator capable of producing an ozone concentration ranging from 18 to 65 Nm^3^ was utilized for obtaining three ozone concentration levels in water: 3, 5 and 10 mg/L. Ozonated water was placed in a 70 L plastic box where 500 g grape samples closed in perforated plastic clamshell containers were immersed utilizing two washing times (5 and 10 min). Overall, six ozonated water treatments were tested. After the ozonated water treatments, all samples were stored for 30 days at 2 °C and 95% relative humidity to simulate commercial practices. The pesticide residue contents were determined before the ozonated water treatments (T_0_) and 30 days after the cold storage (T_1_). The treatments with ozonated water washing reduced the pesticide residues up to 100%, while the SO_2_ control treatment reduced the pesticide residues ranging from 20.7 to 60.7%. Using 3 mg/L ozonated water to wash grapes for 5 min represented the optimal degradation conditions for all of the analyzed pesticides, except for fludioxonil, which degraded better with a washing time of 10 min. The ozone treatments did not significantly reduce the gray mold and the fungal and bacterial microbiome, while a relevant reduction was observed in the yeast population.

## 1. Introduction

Plant protection products (PPPs), or pesticides for short, are widely used against pathogens, insects or weeds to prevent crop damage. It was estimated that 30–40% of food is lost if adequate protection is not provided by PPPs [1]. Therefore, it is essential to provide the minimum level of pesticides that ensure food health and accessibility. For this reason, the application of PPPs and their subsequent degradation have to be investigated. The degradation processes of PPPs are due to dissolution in the surrounding atmosphere, hydrolysis, microbial degradation, oxidation, penetration and photodegradation [2]. Nevertheless, minimal amounts may remain as pesticide residues in food until harvest and reach consumers with possible chronic health effects [3]. Pesticide residues are subject to legal regulation and monitoring. For each active compound, the maximum residue limit (MRL) indicates the legal amount for placement on the market and allows national authorities to verify that PPPs have been used correctly.

Table grapes are among the foods where MRLs are most frequently exceeded [4]. Residues in fresh and processed products are controlled not only via official monitoring by national authorities but also independently by distributors, processing industries, importers and growers through the application of secondary requirements that are becoming more numerous and complex. Initially introduced in the European Union, they now play an increasingly important role in international trade. These requirements may be based on the limits set by law, but they go beyond the legal requirements; in fact, secondary requirements are generally based on a lower percentage of the MRLs set by law and, for certain crops, on a maximum number of detectable residues at the limit of quantification (LOQ) of the analytical method. For this reason, many distribution chains and food industries require growers to reduce residue levels to an even greater extent. Therefore, in addition to controlling PPPs’ preharvest interval time in the field, effective ways are being considered to preventively remove pesticide residues already present on vegetables to avoid adverse effects on human’s health [5].

One of the latest methods involves the generation of ozone gas in water as a washing treatment to reduce pesticide residues in different fruits and vegetables [6,7,8,9,10,11,12]. Ozone is a natural substance in the atmosphere that is generally recognized as safe (GRAS) for food contact applications [13]. Ozone is also an effective sanitizer against a wide range of microorganisms and enables the elimination of mycotoxins [14,15,16,17,18,19]. The impact of ozone on pesticide residues is not always equally effective. Swami et al. [6] found that ozonated water was more efficient than normal water washing for the removal of pesticide residues from grapes and green peppers. By contrast, Sadlo et al. [20] found that ozonated water was no more effective than simple washing processes in reducing pesticide residues on apples. However, the efficiency of ozone treatment in degrading pesticide residues is influenced by several factors, mainly the ozone concentration, the duration of treatment, the type of food, the class of pesticide and the degree of contamination by pesticide residues [21].

Within this context, the present study was conducted to investigate the effectiveness of postharvest washing with ozonated water on reducing pesticide residues in ready-to-eat seedless table grapes. The effects of the treatment on the microbiological aspects were evaluated, both in regard to the control of the gray mold, caused by *Botrytis cinerea* Pers., and the berry microbiome (fungi, bacteria and yeasts). PPPs commonly used in pest control in table grapes were included in the study trial and detected as residues. The analyzed PPPs were three systemic insecticides (Acetamiprid, Flupyradifurone and Spirotetramat), together with five systemic, locally systemic and nonsystemic fungicides (Fludioxonil, Fluxapyroxad, Penconazole, Proquinazid and Trifloxystrobin).

## 2. Materials and Methods

### 2.1. Plant Material, Growth Conditions and Viticulture Management Practices

The study was conducted in 2022 on *Vitis vinifera* L. (cv. Princess^®^ seedless) located in a commercial table grape vineyard growing in a Mediterranean environment [22]. Vines were trained onto a ‘double tendone’ trellis system [23], and they were irrigated by means of two 8 L h^−1^ drippers per vine. The vineyard was covered with plastic film from bud break to harvest.

Plant nutrition and pest and disease control were carried out in accordance with local standards. However, more treatments were added to simulate degradation of more pesticides. In particular, the grapes were treated fifteen days before harvest with acetamiprid, flupyradifurone, spirotetramat, fludioxonil and penconazole and forty days before harvest with fluxapyroxad, proquinazid and trifloxystrobin in order to respect the preharvest interval time of each product. The products were used according to the respective maximum doses indicated on the labels (Table 1).

Starting from the onset of the cell enlargement stage, GA (BERELEX^®^, distributed by Syngenta Crop Protection S.p.A., Milano, Italy) was applied on the vines at different concentrations on the basis of the berry diameter.

The date of the harvest (15 October 2022) was determined on the basis of the commercial ripening, which was fixed at 20 °Brix, 5.5 g/L tartaric acid and 4.5 as TSS, titratable acidity and pH, respectively.

### 2.2. Ozonated Water Washing and Pesticide Residues Determination

**Figure 1:**
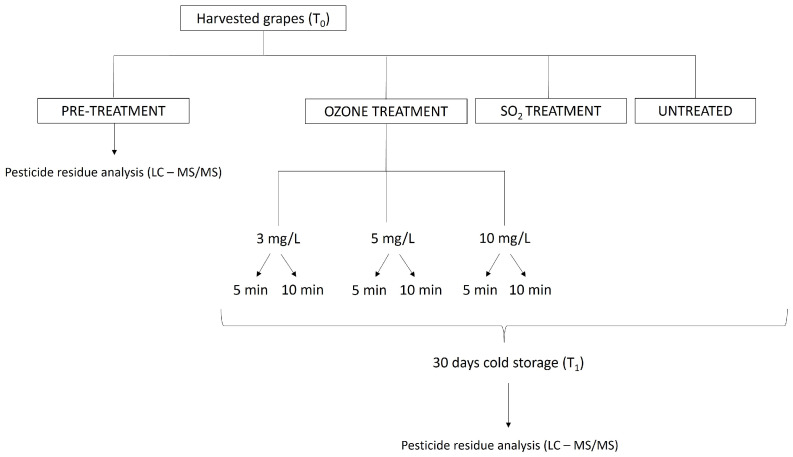
Flow chart of the grape treatment during the experiment.

**Figure 2:**
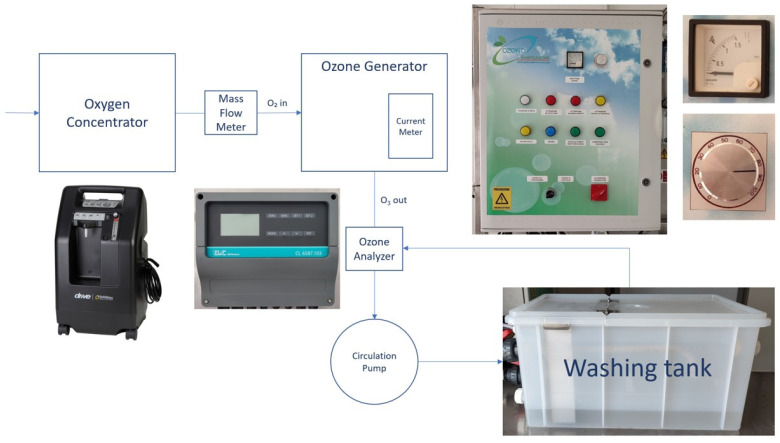
Scheme of the apparatus used for the ozonated water washing of the grapes.

Before the cold storage period (T_0_), a 60 kg grape sample was randomly collected from the harvested grapes and preliminary utilized for picking three replicates of 500 g each for pesticide residues determination (Figure 1). The remaining grapes were utilized to assemble 500 g closed perforated plastic clamshell containers (12 for each treatment) for the ozonated water treatments and subsequently for the cold storage period (Figure 1). The ozonated water treatments were performed at the beginning of the cold storage. A 70 L plastic box containing water was connected to an ozone generator, and it was continuously alimented by ozonated water at different concentrations. The ozone generator was capable of producing ozone concentrations ranging from 18 to 65 Nm^3^, and it was utilized for obtaining three ozone concentration levels in water: 3, 5 and 10 mg/L, which were monitored by an ozone analyzer (Figure 2).

After the ozone concentration reached the fixed level in the water, the 500 g replicate plastic clamshell containers were immersed utilizing two immersion times: 5 and 10 min. Overall, six different ozonated water treatments were provided and immediately destinated to cold storage. Contemporarily, three SO_2_ generating plastic bags containing four 500 g grape samples in the plastic clamshell containers were prepared and stored together with the ozonated samples. To compare all treatments with a control and to test the effect of the cold storage alone, four 500 g grape samples in plastic clamshell containers were prepared and stored without any treatments. All activities were carried out at room temperature (approximately 17 °C), and all samples were stored for 30 days at 2 °C and 95% relative humidity to simulate commercial practices and to take into account the market requirements for the shelf-life cold storage of packaged ready-to-eat table grapes [24]. At the end of the 30-day cold storage period (T_1_), the pesticide residue contents were determined on the three 500 g replicates for each treatment. The pesticide residues determination was performed using the UNI EN 15662:2018 method with LC–MS/MS determination.

### 2.3. Biological Assessment of Gray Mold and Berry Microbiome

**Figure 3:**
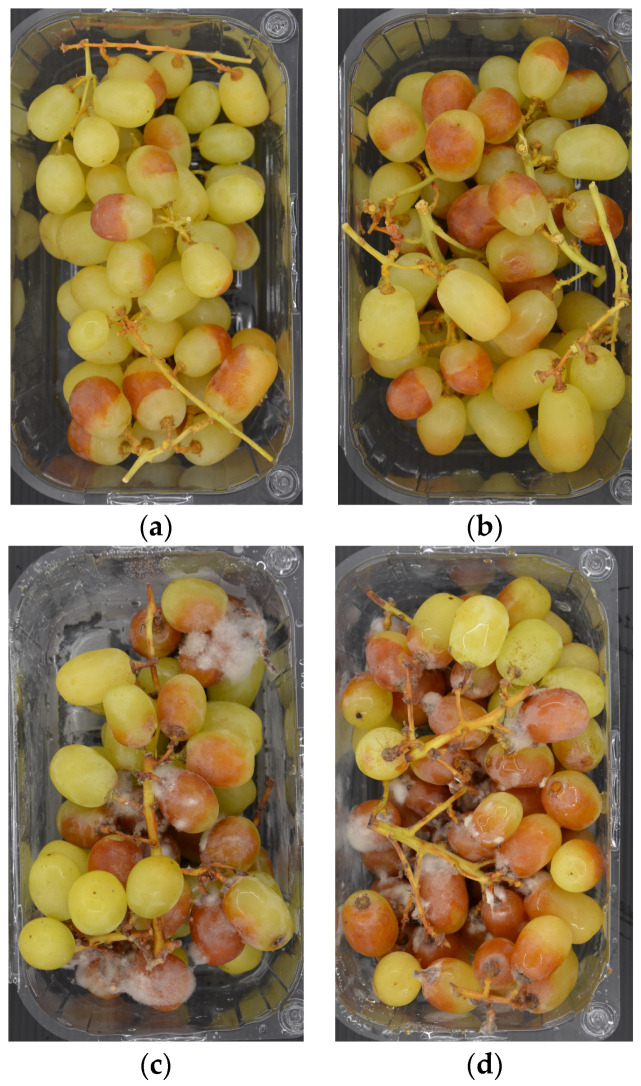
Empirical rating scale from 0 to 4: 0 = no visible symptoms (not represented); (**a**) 5–10% of the berries affected by ‘slip skin’; (**b**) 10–25% of the berries affected by ‘slip skin’; (**c**) 25–50% of the berries affected by ‘slip skin’ and covered by fungus mycelia; (**d**) more than 50% of the berries affected by ‘slip skin’ and covered by fungus mycelia.

After 30 days of cold storage (T_1_), both the incidence and severity of gray mold were evaluated in each treatment. The incidence of gray mold was calculated as the ratio between the number of infected berries and the total number of berries. The severity of gray mold for each plastic container/treatment was evaluated using an empirical rating scale from 0 to 4 considering the presence of fungus mycelia on the berries and the separation of the cuticle from the flesh due to macerating enzymes produced by *B. cinerea* below the skin (‘slip skin’) (Figure 3): 0 = no visible symptoms; 1 = 5–10% of the berries affected by ‘slip skin’; 2 = 10–25% of the berries affected by ‘slip skin’; 3 = 25–50% of the berries affected by ‘slip skin’ and covered by fungus mycelia; 4 = more than 50% of the berries affected by ‘slip skin’ and covered by fungus mycelia. The relative McKinney’s index was also calculated [25].

To evaluate berry microbiome, both at T_0_ and T_1_, an approximately 10 g of berries from each treatment showing no signs of exterior damage was placed in a large beaker containing 200 mL of sterile Ringer solution (NaCl 2.25 g, KCl 0.01 g, CaCl_2_ 0.12 g, NaHCO_3_ 0.05 g and four drops of TWEEN 20) under biological hood conditions. After coating the beaker with aluminum foil, it was put under agitation for 30 min to allow the microorganisms to separate from the skin of the berries. The resultant microbial suspension (mother solution) was serially 1/10 diluted in sterile plastic vials, where 1 mL of the suspension was eluted in 9 mL of sterile Ringer solution three times up to a 10^−3^ CFU mL^−1^ concentration. Appropriate volumes of the diluted microbial suspensions were subsequently plated onto selective solid growth media to evaluate the nature of the epiphytic microbiome: 200 μL on Wallerstein Laboratory (WL) Nutrient Medium (VWR Chemicals, Leuven, Belgium) for yeast populations; 100 μL on NB (NaCl 5 g, meat peptone 5 g, yeast extract 2 g and agar powder 16 g) for bacteria; 500 μL on TSM (KCl 0.151 g, K_2_HPO_4_ 0.9 g, MgSO_4_ 0.2 g, NH_4_NO_3_ 3 g, glucose 3 g, Bengal rose 0.08 g, agar 20 g, ampicillin 790 µL and streptomycin 1 mL) for fungi. The plating process was followed by a 3 days of incubation at 25 °C, when the colonies eventually reached the desired diameter of 0.25 ± 0.05 mm.

### 2.4. Data Analysis

For the collected data and for each pesticide residue, the analysis of variance (ANOVA) was carried out using a one-way model to evaluate the effect of the cold storage period and the effect of SO_2_ with respect to the ozonated water treatments. For each molecule, a two-way model was performed to test the effect of the washing time and ozone concentration in the water. An F test was used to compare averages in the one-way models and to test the factors’ interaction in the two-way model. When interactions were significant, the means were separated with Tukey’s HSD test (*p* < 0.05). The ANOVAs was performed using STATISTICA software v. 6.0 (StatSoft Inc., Tulsa, OK, USA).

## 3. Results

### 3.1. Pesticide Residues’ Detection and Degradation Effects of Ozonated Water

**Figure 4:**
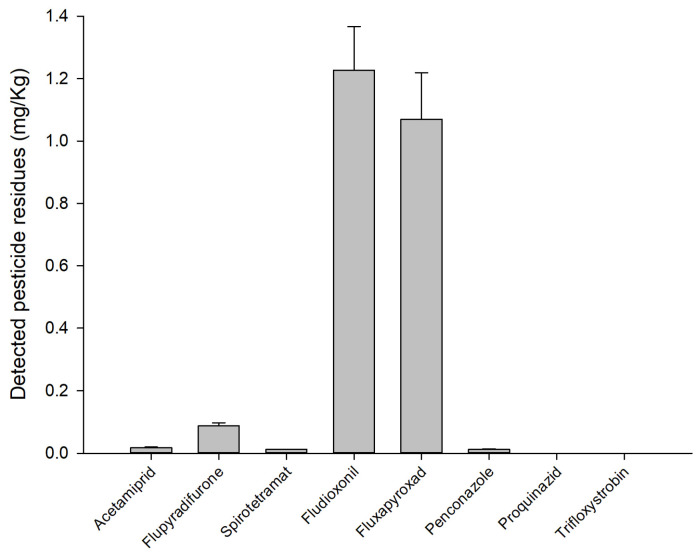
Pesticide residues (mg/kg) detected in the grapes at harvest time (T_0_).

The residues of all three insecticides (acetamiprid, flupyradifurone and spirotetramat) and the five fungicides (fludioxonil, fluxapyroxad, penconazole, proquinazid and trifloxystrobin) were found in different quantities in the samples at time T_0_ (Figure 4). Fludioxonil and fluxapyroxad were detected in major measurements (1.227 ± 0.140 and 1.070 ± 0.149 mg/kg, respectively) (Figure 4). All molecules were lower than the EU MRL (Table 1). Proquinazid and trifloxystrobin were detected at trace levels, namely, smaller than the limit of quantification (LOQ) of the analytical method (<0.005 mg/kg) (Figure 4).

**Figure 5:**
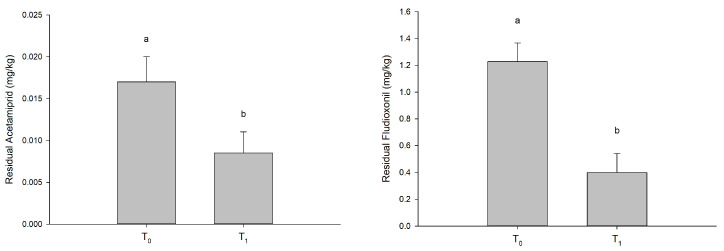

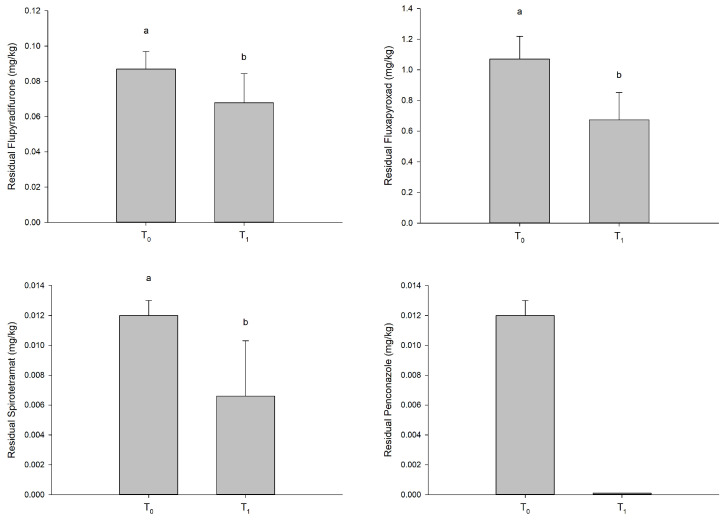
The residual pesticide (mg/kg) values in grapes at harvest time (T_0_) and in untreated grapes after 30 days of cold storage (T_1_). The bars labeled by different letters are significantly different according to Tukey’s test (*p* < 0.05).

Overall, the pesticide residues contents showed a general decreasing trend when comparing at pre- and post-cold storage (T_0_ vs. T_1_, Figure 5).

**Figure 6:**
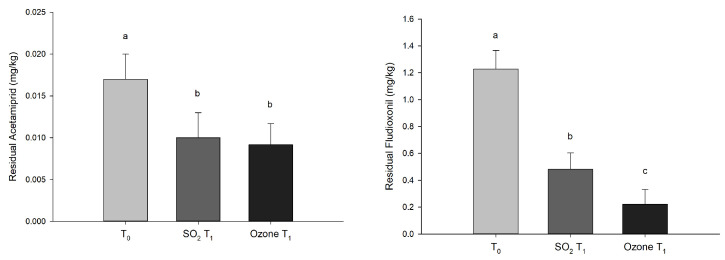

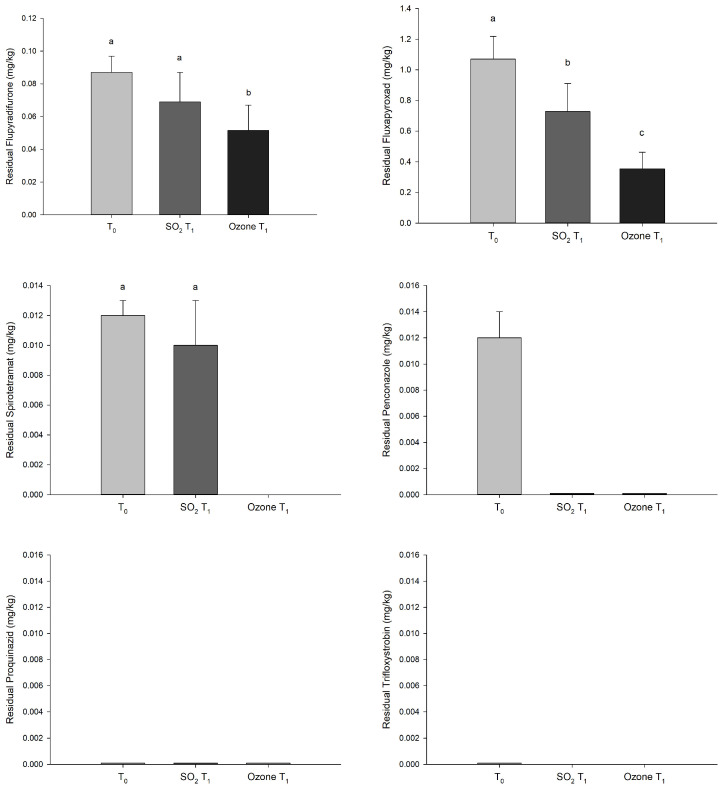
The residual pesticide (mg/kg) values in the grapes at harvest time (T_0_) and after 30 days of cold storage with sulfur dioxide (SO_2_ T_1_) and ozone (Ozone T_1_) treatments. The bars labeled with different letters are significantly different according to Tukey’s test (*p* < 0.05).

The ozonated water treatments showed, in general, better results compared to the SO_2_ treatment (Figure 6). Fludioxonil, which has a contact effect, showed the highest average rate of degradation, which was reduced, on average, by 81.9% in the treatments with ozonated water and by 60.7% in the SO_2_ control treatments. Spirotetramat and fluxapyroxad showed the best results in terms of residues degradation due to the ozone treatment compared to SO_2_: the two systemic molecules were reduced by 100% (trace level < 0.005 mg/kg) and 67%, respectively, compared to the SO_2_ treatment, where reductions of only 16.7% and 32% were observed. Differently, acetamiprid and flupyradifurone showed a low degradation rate, being reduced, on average, by 46.1% and 40.8%, respectively, in the treatments with ozonated water and by 41.2% and 20.7%, respectively, in the SO_2_ control treatments. It should be highlighted that, because of the effects of all of the treatments, only traces (<0.005 mg/kg) of penconazole and proquinazid were detected at T_1_, while trifloxystrobin residues were totally degraded in all of the samples.

**Figure 7:**
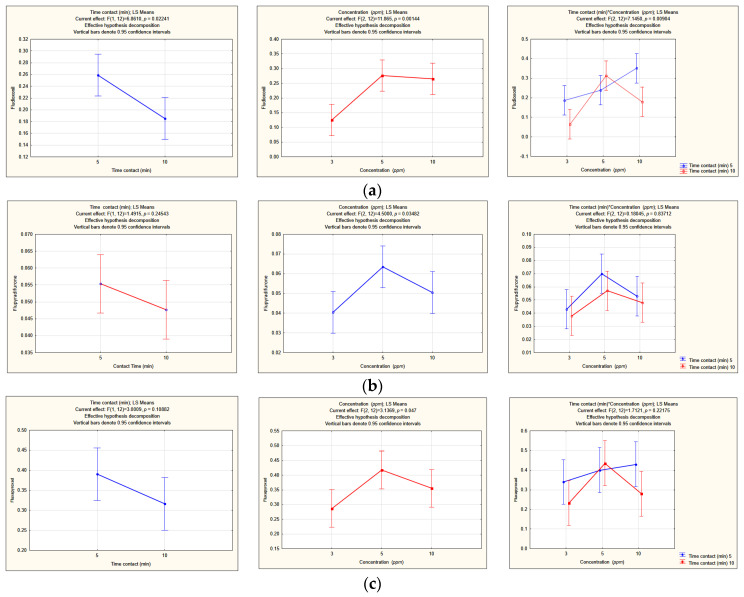
Significant interactions between ozone concentration and washing time: (**a**) fludioxonil; (**b**) flupyradifurone; (**c**) fluxapyroxad. Vertical bars denote 0.95 confidence intervals.

The influence of the ozone concentration and washing time, together with their interactions, on the pesticide residues removal are reported in Table 2. Significant differences in residual fludioxonil were found either because of ozone concentrations (*p* < 0.01) or washing time (*p* < 0.05). A positive interaction between ozone concentration and washing time was observed (*p* < 0.01) (Figure 7a). Significant differences (*p* < 0.05) in flupyradifurone and fluxapyroxad residues were only due to ozone concentration (Figure 7b,c). No significant effect due to the two factors was observed in the other pesticides.

### 3.2. Effects of Ozone Treatment on Gray Mold and Berries Microbiome

In our experiment, the effects of different ozonated water washing on gray mold and the microbiome of berries in semi-commercial postharvest conditions were tested compared to SO_2_ treatment. None of the tested ozonated water treatments was more effective in controlling gray mold compared to the SO_2_ treatment (Table 3).

**Figure 8:**
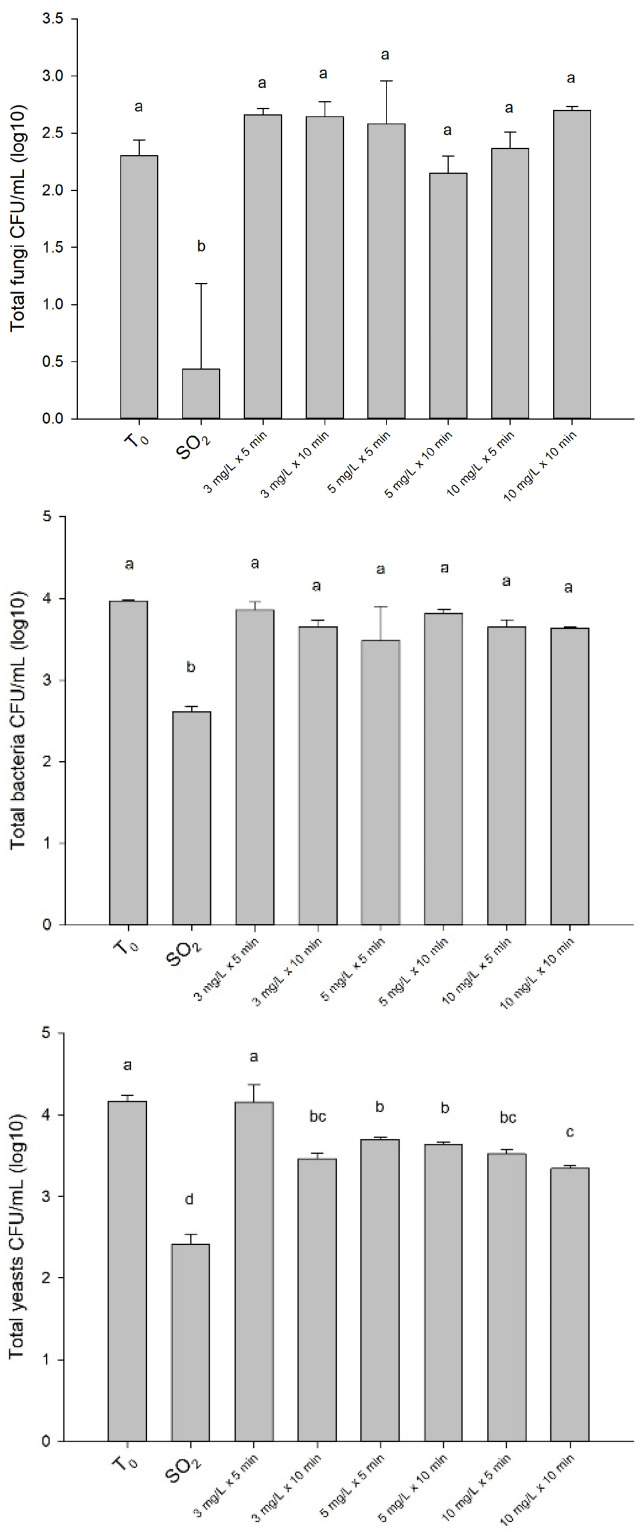
Total fungi, bacteria and yeasts (CFU/mL) before (T_0_) and after 30 days of cold storage with sulfur dioxide (SO_2_) and different ozone treatments. The data are presented as the mean of three replicates with standard deviation. The bars labeled with different letters are significantly different according to Tukey’s test (*p* < 0.05).

Berries treated with SO_2_ showed a McKinney’s Index of 30.6%, while the different tested ozone treatments ranged from 66.7 to 80.6%. The lack of ozone impact on the control of fungi was also confirmed by the results of microbiome analysis (Figure 8). Unlike the SO_2_ treatment, the different tested ozone treatments did not significantly reduce the fungal population initially present on the berries at harvest time (T_0_). The same result was also observed regarding the bacterial microbiome, whose ineffectiveness can be related to a high ozone resistance of the bacterial spores, as reported by Khadre et al. [14]. Finally, relevant differences were observed in yeast’s population by comparing the effects of different ozone treatments vs. SO_2_ treatment. The greatest reduction of yeasts was caused by SO_2_ (−42.0% compared to T_0_). With regard to the different ozone treatments tested, the greatest reduction of yeasts was caused by a long washing time (10 min combined with 10 or 3 mg/L) (−19.7 and −16.8%, respectively) or high ozone dose with a short washing time (10 mg/L × 5 min) (−15.4%). No effect was observed with a minimal ozone dose and minimal washing time (3 mg/L × 5 min).

## 4. Discussion

All three insecticides (acetamiprid, flupyradifurone and spirotetramat) are molecules with systemic properties. Acetamiprid (neonicotinoid) and flupyradifurone (butenolide) are classified as nicotinic acetylcholine receptor (nAChR) competitive modulators, while spirotetramat is an inhibitor of acetyl CoA carboxylase, according to the IRAC classification [26]. According to fungicide FRAC classes [27], penconazole is classified as a DMI-fungicide and shows systemic properties, while fluxapyroxad (SDHI-fungicides), proquinazid (azanaphthalenes) and trifloxystrobin (QoI-fungicides) show locally systemic properties; they are all widely used against powdery mildew. Finally, fludioxonil (phenylpyrroles) is a contact fungicide effective against gray mold.

In our study, fludioxonil showed the highest average rate of degradation: pesticide properties could be considered responsible for this behavior given that the ozonated water could have degraded pesticide residues on the berry surface with a greater efficiency compared to the pesticides absorbed into the tissues [7].

However, in spite of their low water solubility, spirotetramat (systemic) and fluxapyroxad (locally systemic) showed the best results in terms of residues degradation because of the ozone treatment compared to SO_2_. These results suggest that in addition to the cleaning effect of the pesticide residues on berries’ surface the ozonated water may gradually penetrate the first layers of the fruits and act on some pesticides that are absorbed into the first layers of the fruits’ tissues, considering that the impact of ozone is significantly limited once the active substance passes the cellular wall [15]. Therefore, the efficiency of pesticide residue removal is different in various fruits, because their different type surfaces (soft, coarse, smooth, glossy or hard) can affect the absorption and penetration properties [6].

On the contrary, in spite of their high water solubility, acetamiprid (systemic) and flupyradifurone (systemic) showed a low rate of degradation with the ozone treatment. This proves that molecule water solubility alone does not seem to govern the removal of a pesticide. Instead, the very low initial concentration of acetamiprid and flupyradifurone residues was probably one of the factors that inhibited their degradation. This is because the higher initial contamination resulted in faster degradation owing to the higher concentration of the degradable target (reactant); this phenomenon could be attributed to the pesticide degradation kinetics [7].

The best fludioxonil residues degradation was obtained with a minimum ozone dose (3 mg/L) combined with a maximum washing time (10 min), providing a reduction of 94.8% (Figure 7a). Regarding flupyradifurone and fluxapyroxad, the minimum ozone dose (3 mg/L) showed a better removal efficiency and independently from the washing time (Figure 7b,c). The higher residues of flupyradifurone with 5 and 10 mg/L ozone concentrations could be attributed not to a lower efficiency of these ozone treatments in respect to the 3 mg/L dose but to the pesticide accumulation in the washing water; indeed, this active substance has a high water solubility, and it is quite stable in water because of its high water DT50 (Table 1). So, according to Sadlo et al. [20], the pesticide may have transferred from the contaminated water to the grape clusters during the washing, since in our test the treatments were performed in succession starting from the lowest dose. The same could have occurred for fludioxonil and fluxapyroxad, despite their lower water solubility and moderately fast water DT50 (Table 1).

On the basis of these results and considering the time, cost and treatment efficiency, it could be concluded that using 3 mg/L ozonated water to wash grapes for 5 min represents the optimal degradation conditions for all of the analyzed pesticides, except for fludioxonil, which degrades better with a 10 min washing time. Consequently, the results supported by the trials carried out may fit and be consistent with the usual commercial practices. Finally, given that high ozone concentrations could likely affect human health and cause corrosion [28], it has to be taken into account that a concentration of 3 mg/L could be relatively safer for humans, as well as for vegetables. A potential health risk could be linked to pesticide degradation byproducts caused by ozonated water treatments given that they may be more toxic than the pesticides themselves. In this regard, earlier studies have already indicated that only traces of unstable degradation byproducts could be found and that no bioaccumulation and toxicity were detected when ozone was used to degrade pesticides [5,29]. However, further research should be undertaken to investigate the toxicity of byproducts resulting from the ozonated water treatment of the tested pesticides.

Moreover, it may be useful to continue to apply ozone to waste water after the described treatments in order to remove pesticide residues in it. Reports on the degradation of pesticides in water using ozone or other oxidants are available [5,30,31], so it is expected that continuous ozone treatment may be able to break down the molecules accumulated in waste water, thus solving the issue of waste water disposal.

Ozone treatment is unlikely to replace sulphur dioxide treatment as a means of controlling gray mold of table grapes. Therefore, if this method were to be used commercially, it is likely that packers would follow the ozone treatment with SO_2_, with possible additional benefits in terms of reducing pesticide residues. The effect of using these techniques in combination should be further investigated.

Cleaning grapes with ozonated water can also bring about a decrease in yeast populations on berries, and this could play an important role in counteracting the onset of alterations caused by non-*Saccharomyces* yeasts, preserving grape health; this could represent an element for further analysis if applied to grapes destined for winemaking.

Ozonation treatment could offer many advantages in degrading pesticide residues, including ease of use, relatively low cost and user safety. In addition, ozone decomposes into oxygen without producing any additional traces of byproducts. To conclude, fruit safety and quality may benefit from the use of ozonated water washing as part of the postharvest treatment of table grapes.

## Figures and Tables

**Table 1:** Activity spectra, mobility, water solubility (W.S.), water DT50 (W.DT50), soil DT50 (S.DT50) and MRL of grapefruits in the European Union of the insecticides and fungicides studied.

Molecule (Commercial Product, Dose)	Activity Spectra	Mobility	W.S. (mg/L)	W.DT50 (Days)	S.DT50 (Days)	MRL (mg/Kg)
Acetamiprid (Epik SL, 2 L/ha)	Aphids, whiteflies, thrips	Systemic	2950	4.7	3	0.5
Flupyradifurone (Sivanto Prime, 0.5 L/ha)	Aphids, leafhoppers	Systemic	3200	31.4	130	3
Spirotetramat (Movento 48 SC, 1.5 L/ha)	Aphids, mealybugs	Systemic	29.9	0.8	0.7	2
Fludioxonil (Geoxe, 1 kg/ha)	Gray mold	Contact	1.8	2	16	5
Fluxapyroxad (Sercadis, 0.15 L/ha)	Powdery mildew	Locally systemic	3.4	4.4	181.5	3
Penconazole (Scudex, 0.2 L/ha)	Powdery mildew	Systemic	73	2	89.7	0.5
Proquinazid (Talendo, 0.2 L/ha)	Powdery mildew	Locally systemic	0.9	0.8	30.5	0.5
Trifloxystrobin (Flint, 0.15 kg/ha)	Powdery mildew	Locally systemic	0.6	1.1	1.7	3

**Table 2:** Residual pesticide as affected by ozone concentration, washing time and their interaction.

Factors	Acetamiprid	s.e.	Flupyradifurone	s.e.	Fludioxonil	s.e.	Fluxapyroxad	s.e.
*Ozone concentration*								
3 mg/L	0.009	±0.001	0.041 b	±0.005	0.126 b	±0.024	0.287 b	±0.037
5 mg/L	0.010	±0.001	0.064 a	±0.005	0.276 a	±0.024	0.418 a	±0.037
10 mg/L	0.010	±0.001	0.051 a	±0.005	0.265 a	±0.024	0.355 a	±0.037
Significance	n.s.		*		**		*	
*Washing time*								
5 min	0.010	±0.001	0.055	±0.004	0.259 a	±0.020	0.390	±0.030
10 min	0.008	±0.001	0.048	±0.004	0.185 b	±0.020	0.316	±0.030
Significance	n.s.		n.s.		*		n.s.	
*Interactions*								
Ozone concentration× Washing time	n.s.		n.s.		**		n.s.	

* *p* < 0.05, ** *p* < 0.01, n.s., Not significant. Different letters in the columns denote significant differences according to Tukey’s test.

**Table 3:** McKinney’s Index of gray mold in grapes treated with SO_2_ and different ozone treatment after 30 days cold storage.

Treatment	McKinney’s Index (%)
Sulfur dioxide	30.6 b *
10 mg/L × 10 min	80.6 a
10 mg/L × 5 min	80.6 a
5 mg/L × 10 min	80.6 a
5 mg/L × 5 min	77.8 a
3 mg/L × 10 min	77.8 a
3 mg/L × 5 min	66.7 a

* Different letters indicate significant differences according to Tukey’s test (*p* < 0.05).

## Data Availability

Data is contained within the article.
